# Major depression disorder may causally associate with the increased atrial fibrillation risk: evidence from two-sample mendelian randomization analyses

**DOI:** 10.1186/s12920-023-01565-0

**Published:** 2023-06-23

**Authors:** Lei Wang, Chunhua Ding

**Affiliations:** Cardiac Department, Aerospace Center Hospital, 15 Yuquan Road, Haidian District, Beijing, 100049 PR China

**Keywords:** Major depression disorder, Atrial fibrillation, Mendelian randomization analyses

## Abstract

**Background:**

Observational studies have revealed a link between major depressive disorder (MDD) and a higher chance of developing atrial fibrillation (AF). It is still uncertain whether or not this correlation indicates a causal relationship. This research set out to evaluate the causal impact of MDD on AF.

**Methods:**

To evaluate the causal relationship between MDD and AF, we employed a two-sample Mendelian randomization (MR) method. A new genome-wide association study (GWAS) with 500,199 participants was used to obtain an overview of the association of genetic variations with MDD. An additional GWAS incorporating 1,030,836 people provided data on the relationship between gene variants and AF. The inverse-variance weighted technique was utilized to assess the effect sizes. Sensitivity analysis included the use of other statistical approaches such as weighted median, Outlier, MR Pleiotropy Residual Sum, weighted mode, simple mode, and MR - Egger.

**Results:**

By employing 47 single nucleotide polymorphisms (SNPs) as markers, MR analyses in random-effect inverse-variance weighted models found that genetically projected MDD was linked to an elevated incidence of AF (odds ratio [OR] = 1.098, 95% CI 1.000–1.206; P = 0.049). No gene pleiotropy was discovered as indicated by MR-Egger (intercept= -0.011, *P* = 0.169). Sensitivity analysis employing other MR techniques yielded reliable results.

**Conclusion:**

This MR study established a causal relationship between genetically predicted MDD and an elevated risk of AF.

**Supplementary Information:**

The online version contains supplementary material available at 10.1186/s12920-023-01565-0.

## Background

Currently, 2–3% of the global population has atrial fibrillation (AF), making it the most prevalent arrhythmia, and this number is predicted to rise worldwide as the population ages [1]. Many risk factors are associated with AF, including chronic diseases such as chronic kidney disease, valvular heart disease, thyroid disease, obesity, sleep apnea, heart surgery, smoking, and so on. AF can lead to stroke, heart failure, and other poor prognoses [1]. Additional possible modifiable risk factors, including mental problems, ought to get greater attention to even further minimize the AF burden.

Major depressive disorder (MDD), sometimes known simply as depression, is hallmarked by long-term sad mood. MDD ranks first among all mental health diagnoses and is linked to a significant risk of death. It is also closely linked to cardiovascular disease [2–5]. High rates of depression have been shown in individuals with AF, with 9.2% of those experiencing symptoms of AF being diagnosed with MDD [6]. There was a 25-34% greater chance of developing AF in those with MDD [7, 8]. MDD is correlated with both the onset and progression of AF, which complicates therapy and elevates the risk of unfavorable consequences in AF patients [9, 10]. Recent research has also linked MDD to an elevated risk of AF recurrence in individuals after ablation [11]. Although accumulating observational evidence has demonstrated that major depressive disorder was associated with development of atrial fibrillation, the causal effect of major depressive disorder on atrial fibrillation is still unclear because of the possibility of biases such as confounding or reverse causality.

Mendelian randomization (MR) applying instrumental variable (IV) techniques to estimate causal relationships between genetic risk factors and complex human traits is an increasingly popular method [12]. Because exposed IVs are randomly assigned during conception and are not expected to be influenced by disease state, MR studies may assess the causality between exposure and illness outcome by eliminating unobserved confounders and reverse causality [13]. If an exposure like MDD can directly affect an outcome like AF, then variants that affect MDD should affect AF to some proportional extent. Nonetheless, due to horizontal pleiotropy, it is necessary to rule out other mechanisms via which such variations may impact AF. In this investigation, we used data from a major genome-wide association study (GWAS) involving MDD and AF to undertake a two-sample MR and demonstrate the direct causal relationship between MDD and the risk of AF.

## Methods

**Fig. 1:**
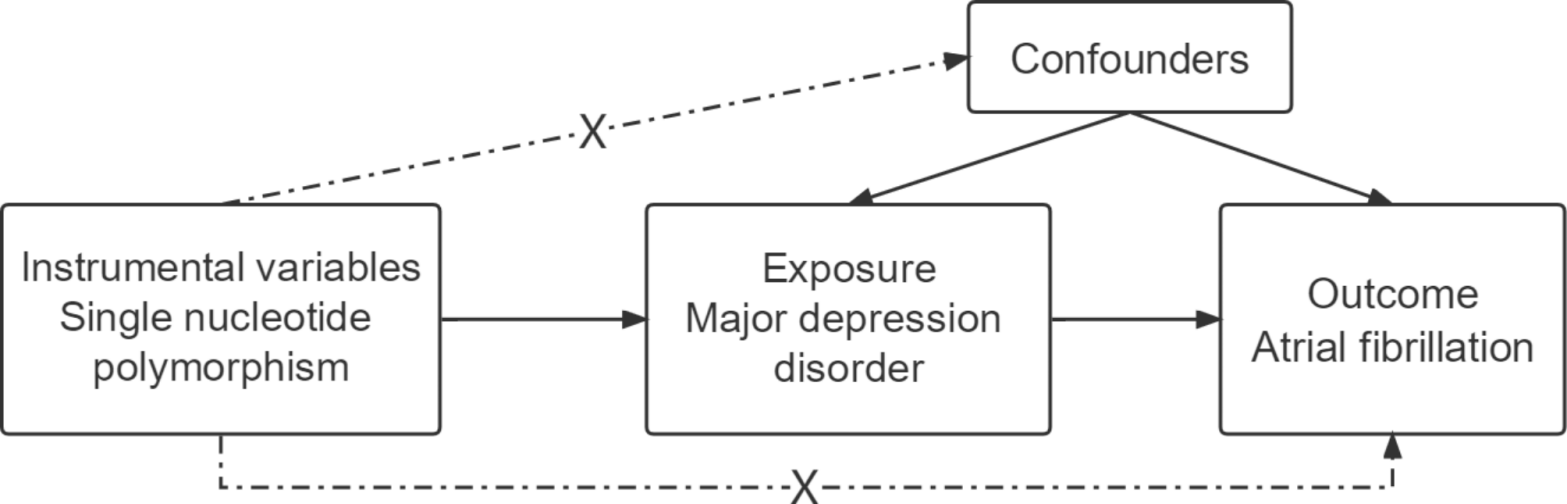
Flow chart of the analyses and core hypotheses for the two-sample Mendelian Randomization (MR) study. MR model of major depressive disorder (MDD) and risk of atrial fibrillation. The approach is based on the hypothesis that the genetic variations impact solely through MDD and are linked to the condition itself but not to confounders

To assess the causal relationship between MDD and AF risk, we used a two-sample MR analysis predicated on publicly available summary-level datasets from GWASs [12]. Each cohort that participated in the GWAS was subjected to ethical approval and participant consent, and the summary-level information was made available for analysis. The causal effects of MDD on AF were investigated via a two-sample MR method (Fig. 1).

### GWAS summary data for MDD

For MDD, we use psychiatric genomics alliance (PGC) major depression GWAS summary statistics. Participants were drawn from two cohorts in this research (UK Biobank and PGC). The study included 500,199 individuals (170,756 patients with MDD and 329,443 controls) [14]. By relying on patient reports, clinician evaluations, and reviews of medical records, the study employed a broad definition of MDD. Genes and genetic pathways implicated in synaptic morphology and neurotransmission were among those found to be related to depression in this research, along with 102 independent variants, 15 gene sets, and 269 genes. Fifty single-nucleotide polymorphisms (SNPs) were shown to be strongly linked to MDD (p < 5 × 10^− 8^), and the use of a two-sample MR technique necessitates that the instrument is free of linkage disequilibrium (LD) with one another (r^2^ < 0.001, distance threshold > 10,000 kb). Table S1 has a detail of the fifty SNPs. The variability in MDD was explained by these 50 SNPs to the extent of 0.35% [15].

In the MR analysis, there may be an increase in the weak instrumental bias due to the overlapping of sample participants utilized to determine the genetic link between exposure and outcomes. To evaluate the strength of the exposure IVs, we calculated the F-statistic of SNP. The instruments’ F-statistic of 35 was much higher than the conventional value of 10, showing a high predictive ability for MDD [16]. The below equation was used to compute R^2^= (2 × EAF [1- EAF] × β^2^), with EAF representing the effect allele frequency and β representing the estimated genetic influence on MDD. To evaluate the strength of each SNP, we computed the F-statistic premised on the equation below: F = (R^2^ × [n − 1 − k])/ ([1 − R^2^] × k) as indicated, where, R^2^ was used to determine how much of the phenotypic diversity could be attributed to the underlying genetic variants, k denotes the total count of SNPs, and n represents the size of the sample [17, 18].

### GWAS summary data for AF

To eliminate demographic heterogeneity, only summarized statistics from the Europeans were used. Genes for AF were retrieved from a massive European GWAS Meta-analysis, which compiled data from 1,030,836 individuals, of which 60,620 had AF and 970,216 served as controls [20]. The sample included participants of European descent from six different studies (the AFGen Consortium, UK Biobank, DiscovEHR, Michigan Genomics Initiative (MGI), deCODE, and Nord-Trøndelag Health Study (HUNT)). Cases and controls having genotype data were identified using a mix of inpatient, outpatient, and emergency department discharge diagnoses (ICD-9 and ICD-10). Independent risk variants were found in 142 genes across 111 genomic regions, and 151 functional candidate genes were ranked for their potential roles in AF. Other resources include information on quality assurance, imputation, and genotyping [19].

When a specific exposure SNP is missing from outcome data, LD tagging is not employed to substitute proxy SNPs. Summary data for each of the 49 SNPs linked to MDD were collected from the GWAS Meta-analysis. The rs35469634 SNP was not found in the AF GWAS summary data.

In two-sample MR, it’s crucial to check that the SNPs’ influences on exposure are consistent with the alleles’ effects on outcomes. The SNPs in the two GWAS results were judged to be consistent according to allele frequency, and if inconsistent, the SNPs were removed. Two of the 49 SNPs, rs2876520 and rs4730387, were eliminated because they were palindromic and had a frequency of intermediate alleles.

### Testing mendelian randomization assumptions

MR studies require fulfillment of three core hypotheses [13]: [1] There is a strong relationship between genetic variation and exposure; [2] There was no association between genetic variation and possible confounding factors; [3] Except for the mode of exposure, genetic variation was not associated with the outcome (Fig. 1). Testing hypotheses 2 and 3 is complicated by the presence of unknown potential confounding variables. Because of this, we estimated MR Egger’s regression coefficients and tested for a significant intercept to determine if horizontal pleiotropy was present, i.e., genetics impacted AF in addition to MDD.

### Statistical analyses

Estimating the causality impact of each IV was performed using the Wald ratio, which was determined by dividing the Beta of the relevant SNP in the outcome datasets by the Beta of the same SNP in the exposure datasets.

When Cochran’s Q value was significant (P < 0.05), we employed an inverse-variance weighted (IVW)-based multiplicative random-effects model [20]. In all other cases, we adopted a fixed-effects model. The main analysis used the IVW technique to assess the connection between genetically predicted MDD-associated features and the AF risk. In a meta-analysis of Wald ratios for each SNP, IVW assumed that the only way the tool could have affected the findings was via the exposure of interest.

The combined findings of single and multiple SNP analyses were shown using forest and scatter plots. The single and the multiple SNP effect estimates, corresponding to the 95% confidence intervals, are displayed next to each other in a forest-like figure. The estimated regression lines from the multiple SNP analysis are added in the scatterplot, which compares the single SNP impact on exposure and outcomes (with corresponding standard deviations in both directions).

Our MR study has 100% power to identify an OR of 1.10 for AF per odds of MDD, premised on a sample size of 500,199 and an alpha of 5% [21].

Two Sample MR (version 0.5.6) [22] and MRPRESSO (version 1.0) in R (version 4.1.2) were employed to conduct the analysis. ORs with 95% CIs are used to describe the strength of the link between predicted genetic risk for MDD and the risk of AF. We concluded that the causal results of multiple MR methods were consistent in direction and magnitude (see below) and passed nominal significance in IVW. It was determined that p < 0.001 (0.05/47) provided statistically significant proof of a causal relationship. P-values < 0.05 but above the Bonferroni correction threshold were considered suggestive evidence of potential causality.

### Sensitivity analysis

To determine how robust the correlations were, we conducted a series of sensitivity analyses. We first applied a weighted median method to evaluate the correlations, supposing that a minimum of 50% of the weights were from valid instruments [23]. In addition, the MR-Egger, simple mode, and weighted mode approaches were utilized since they can provide more accurate estimates over a broader variety of conditions, but they are less efficient (wider CIs). We also assessed evidence of horizontal multiplicity by examining MR-Egger intercept data and performing Mendelian randomized multiplicity residual sums and outliers (MR-PRESSO) analysis [24]. We also conducted a leave-one-out analysis using the IVW MR approach, in which each SNP was individually removed from the analysis to investigate the possible effect of outliers and/or pleiotropic SNPs. In addition, we tested the heterogeneity via funnel plots.

## Results

**Fig. 2:**
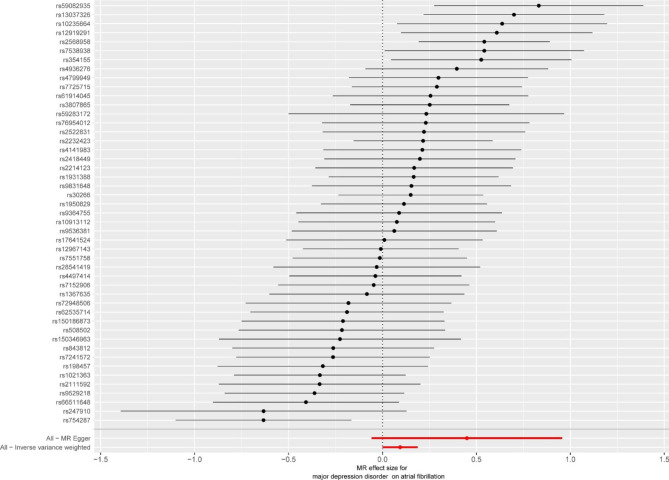
The forest plot of Mendelian Randomization analyses Mendelian Randomization effect size for major depressive disorder on atrial fibrillation for individual variants, MR-Egger and Inverse Variance Weighted

**Fig. 3:**
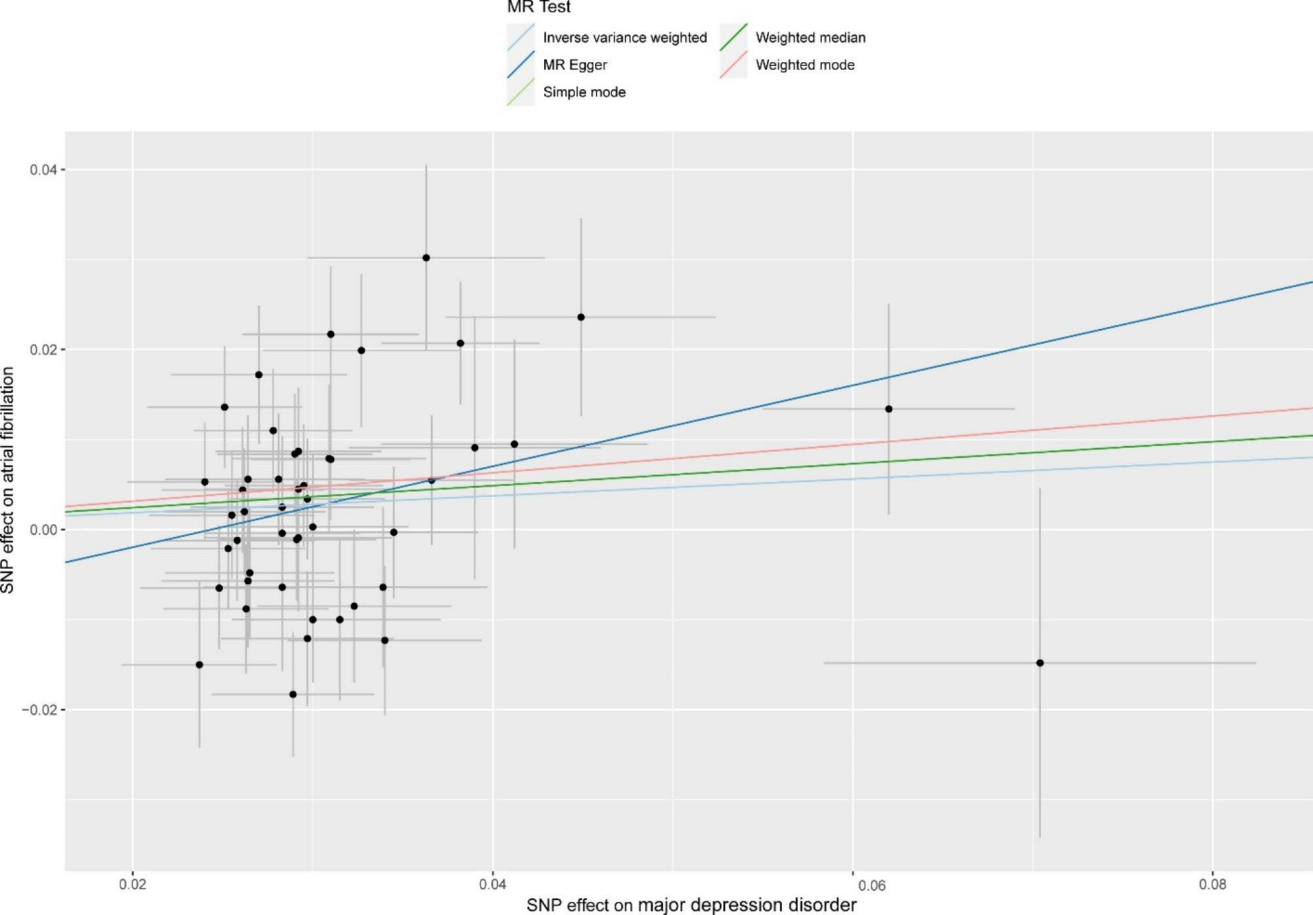
The scatter plot of Mendelian Randomization analyses

The link between genetically predicted MDD-related features and the AF risk was assessed using the random-effects IVW technique since Cochran’s Q value was significant (P = 0.003). As shown in Table 1, in conventional MR analysis by IVW method, we detected the evidence for a potential causal relationship between MDD and AF (odds ratio [OR]: 1.098, 95% CI 1.000–1.206; *P* = 0.049). The forest plot (Fig. 2) and scatter plot (Fig. 3) both displayed the causal estimates deduced from individual IVs .

**Table 1:** Association between MDD and AF by Mendelian Randomization Models

Method	nsnp	OR	95%CI	*P*-Value
Inverse variance weighted	47	1.098	1.000-1.206	0.049
Weighted median	47	1.130	1.014–1.260	0.027
MR Egger	47	1.568	0.944–2.604	0.089
Simple mode	47	1.171	0.903–1.519	0.241
Weighted mode	47	1.171	0.920–1.490	0.206

Abbreviations: CI, confidence intervals; SNP, single nucleotide polymorphism; MR, Mendelian Randomization; OR, odds ratio

Using the weighted median model, we found that MDD and AF had a causal relationship (OR = 1.130, 95% CI = 1.014–1.260; P = 0.027). Results were consistent in MR Egger, weighted mode, and simple mode methods (P > 0.05).

**Fig. 4:**
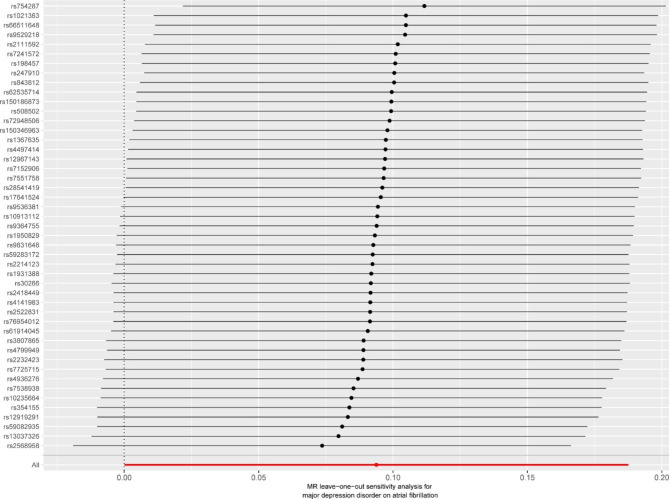
Mendelian Randomization leave-one-out analyses

**Fig. 5:**
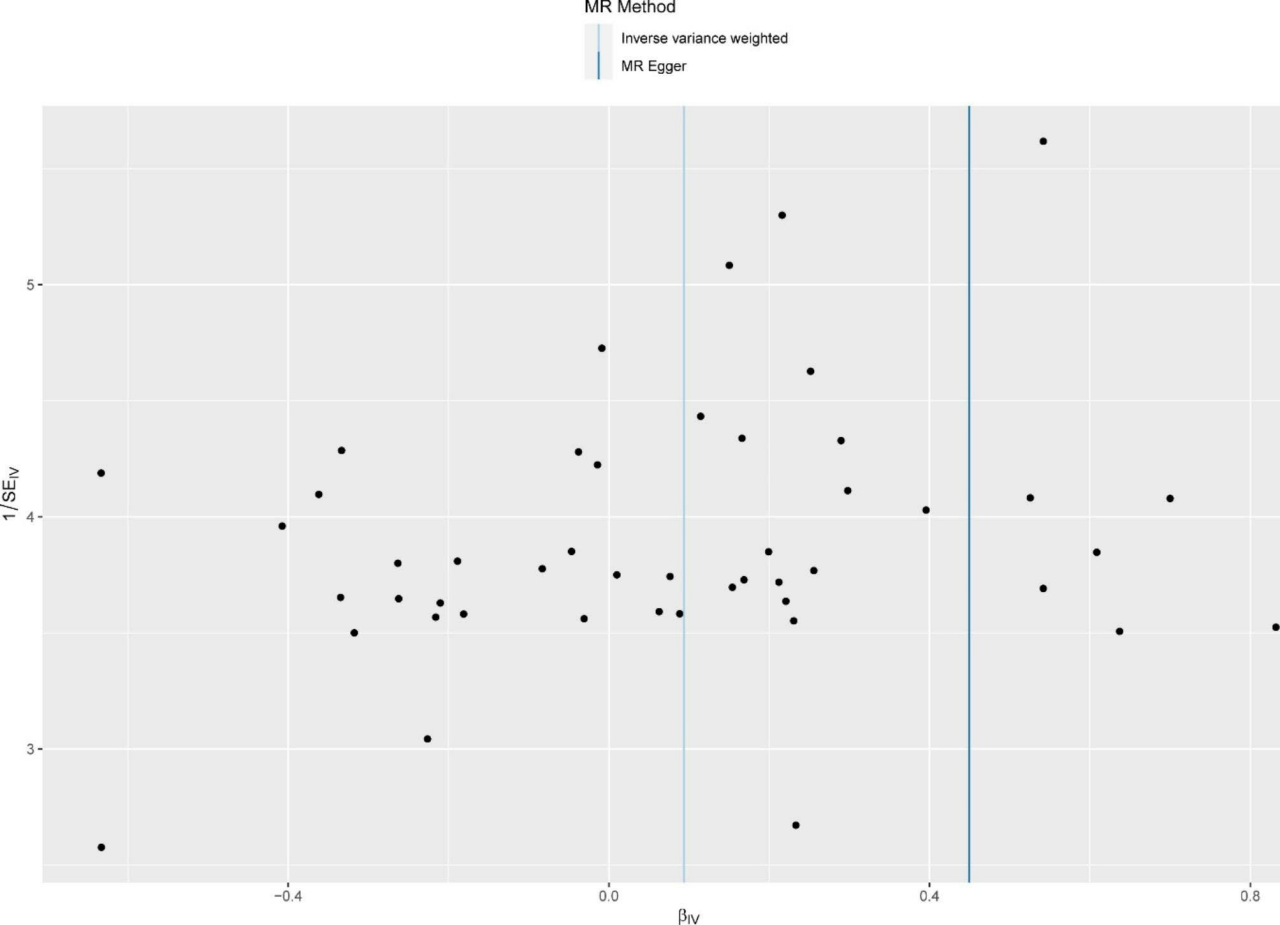
The funnel plot of Mendelian Randomization analyses

There was no pleiotropy (MR-Egger regression test, intercept = -0.011, *P* = 0.169). The MR-PRESSO method identified no variant was excluded from the analyses and no evidence of pleiotropy(*P* = 0.073). We discovered that the overall impact of MDD on AF could not be attributed to a single instrument using a leave-one-out analysis (Fig. 4). Significant heterogeneity was noted for the causal estimates between IVs as per the MR Egger model (Fig. 5).

## Discussion

We performed a two-sample MR analysis using data from publicly available GWASs to evaluate the association between MDD and AF risk and make inferences about their causal relationship. Genetic susceptibility to MDD was associated with an increased likelihood of developing AF, as shown by the statistical causal relationship.

Although several past observational studies found a link between MDD and AF, the findings were inconsistent [8]. Anxiety and depressive symptoms were not linked to an increased incidence of AF according to the findings of a large population-based investigation [25]. Furthermore, antidepressant usage has been linked to an increased incidence of AF [26, 27]. However, given the potential for bias in observational studies attributable to many confounding factors, the causality between MDD and AF is unclear. The assessed AF cases may also have been affected by the fact that those with depression were less likely to comply with treatment [28]. Establishing a causative link between MDD and AF using conventional epidemiological methods is challenging. In contrast with these conventional epidemiological studies, we employed a two-sample MR, a technique that controls for confounding factors by using genetic variants that do not affect the results via biological pathways different from the exposure of interest. After conducting several sensitivity analyses to examine the impact of pleiotropy on causality estimates, we found that our findings remained stable across tests. Researchers Yunlong Lu et al., who conducted the large prospective cohort research, used data from the Atrial Fibrillation Haplotype Reference Consortium (65,446 cases; 522,744 controls), but no link between genetic depression and AF was identified (OR, 1.00; 95% CI, 0.94– 1.06; P = 0.95) [29]. Selection bias as well as the use of a small sample size might explain the discrepancy. Current evidence on antidepressive therapy in patients with AF is limited. These findings need to be corroborated by further research methods, such as large-scale intervention trials and prospective cohort studies.

Our two-sample MR investigation lends credence to the hypothesis that depression might elevate the risk of developing AF. The possible positive effect of MDD on AF has been proposed to be mediated by a myriad of distinct biological pathways. Proinflammatory mediators, particularly interleukin (IL)-2, IL-6, IL-12, and tumor necrosis factor-alpha (TNF-α) have been linked to the progression of depression in past studies [30–32]. The infiltration of inflammatory cells has been seen in the atrium of AF patients, suggesting that AF may be associated with systemic inflammation induced by these cytokines [33, 34]. Moreover, depression may lead to oxidative stress [35, 36], and dysfunction in the autonomic nervous system [37, 38] which could increase the risk for AF [39, 40]. Additional investigation is necessary to understand the molecular processes driving these relationships to enable physicians and researchers to create innovative preventative and therapeutic techniques.

Our findings may influence health care policies for depression and AF. Given the high prevalence of depression and AF in the general population, revealing causality between depression and AF influences public health policies about early prevention and timely intervention. Our finding implied that strengthening screening AF in patients with genetically predicted depression may be necessary. More attention should be paid to reveal association between depression and prognosis of AF. These findings support detection and treatment of major depressive disorder for preventing AF.

### Limitation

This MR study also has limitations. First, these finding needs to be verified in other racial groups to ensure it is applicable beyond Europeans since it was derived using GWAS data of European descent. Second, the study data cannot distinguish between episodes of AF (paroxysmal, persistent, and permanent AF and atrial flutter), conditions that may differ in etiology. Third, estimating the degree of overlap between the exposure samples and the outcome samples was challenging.

## Conclusion

The MR analysis revealed a causal relationship between MDD and elevated AF incidence, a finding that lends credence to the concept of initiating early care and intervention in individuals suffering from MDD to lower their risk of developing AF. Nevertheless, the findings need to be validated via the use of other research schemes, such as large-scale intervention trials and prospective cohort studies.

### What is New?

Although accumulating observational evidence has demonstrated that major depressive disorder was associated with development of atrial fibrillation, the causal effect of major depressive disorder on atrial fibrillation is still unclear.

This study provides genetic evidence of causal effect of major depressive disorder on atrial fibrillation.

### What are the clinical implications?

These findings support detection and treatment of major depressive disorder for preventing atrial fibrillation.

## Electronic supplementary material

Below is the link to the electronic supplementary material.

Supplementary Material 1

## Data Availability

The data that support the findings of this study are openly available in the IEU GWAS database (https://gwas.mrcieu.ac.uk) that comprises the harmonised complete GWAS summary datasets, which can be queried using the R package of TwoSampleMR.

## References

[1] Rahman F, Kwan GF, Benjamin EJ (2014). Global epidemiology of atrial fibrillation. Nat Rev Cardiol.

[2] Li GH, Cheung CL, Chung AK, Cheung BM, Wong IC, Fok MLY (2022). Evaluation of bi-directional causal association between depression and cardiovascular diseases: a mendelian randomization study. Psychol Med.

[3] Zhang M, Chen J, Yin Z, Wang L, Peng L (2021). The association between depression and metabolic syndrome and its components: a bidirectional two-sample mendelian randomization study. Transl Psychiatry.

[4] Zhang F, Cao H, Baranova A (2021). Shared Genetic Liability and Causal Associations between Major Depressive Disorder and Cardiovascular Diseases. Front Cardiovasc Med.

[5] de Geus EJC (2021). Mendelian randomization supports a Causal Effect of Depression on Cardiovascular Disease as the Main source of their comorbidity. J Am Heart Assoc.

[6] Senoo K, Yukawa A, Ohkura T, Iwakoshi H, Nishimura T, Teramukai S (2022). Depression and quality of life in older adults with atrial fibrillation: a cross-sectional community-based study. Geriatr Gerontol Int.

[7] Kim YG, Lee KN, Han KD, Han KM, Min K, Choi HY (2022). Association of Depression with Atrial Fibrillation in south korean adults. JAMA Netw Open.

[8] Garg PK, O’Neal WT, Diez-Roux AV, Alonso A, Soliman EZ, Heckbert S (2019). Negative affect and risk of Atrial Fibrillation: MESA. J Am Heart Assoc.

[9] Ai Y, Xing Y, Yan L, Ma D, Gao A, Xu Q (2022). Atrial fibrillation and depression: a bibliometric analysis from 2001 to 2021. Front Cardiovasc Med.

[10] Baumgartner C, Fan D, Fang MC, Singer DE, Witt DM, Schmelzer JR et al. Anxiety, Depression, and adverse clinical outcomes in patients with Atrial Fibrillation starting warfarin: Cardiovascular Research Network WAVE Study. J Am Heart Assoc. 2018;7(8). 10.1161/JAHA.117.007814 PMC6015441 29656278

[11] Zhuo C, Ji F, Lin X, Jiang D, Wang L, Tian H (2020). Depression and recurrence of atrial fibrillation after catheter ablation: a meta-analysis of cohort studies. J Affect Disord.

[12] Skrivankova VW, Richmond RC, Woolf BAR, Yarmolinsky J, Davies NM, Swanson SA (2021). Strengthening the reporting of Observational Studies in Epidemiology using mendelian randomization: the STROBE-MR Statement. JAMA.

[13] Burgess S, Davey Smith G, Davies NM, Dudbridge F, Gill D, Glymour MM (2019). Guidelines for performing mendelian randomization investigations. Wellcome Open Res.

[14] Howard DM, Adams MJ, Clarke TK, Hafferty JD, Gibson J, Shirali M (2019). Genome-wide meta-analysis of depression identifies 102 independent variants and highlights the importance of the prefrontal brain regions. Nat Neurosci.

[15] Papadimitriou N, Dimou N, Tsilidis KK, Banbury B, Martin RM, Lewis SJ (2020). Physical activity and risks of breast and colorectal cancer: a mendelian randomisation analysis. Nat Commun.

[16] Pierce BL, Ahsan H, Vanderweele TJ (2011). Power and instrument strength requirements for mendelian randomization studies using multiple genetic variants. Int J Epidemiol.

[17] Burgess S, Davies NM, Thompson SG (2016). Bias due to participant overlap in two-sample mendelian randomization. Genet Epidemiol.

[18] Burgess S, Thompson SG, Collaboration CCG (2011). Avoiding bias from weak instruments in mendelian randomization studies. Int J Epidemiol.

[19] Nielsen JB, Thorolfsdottir RB, Fritsche LG, Zhou W, Skov MW, Graham SE (2018). Biobank-driven genomic discovery yields new insight into atrial fibrillation biology. Nat Genet.

[20] Yoshikawa M, Asaba K, Nakayama T (2021). Causal effect of atrial fibrillation/flutter on chronic kidney disease: a bidirectional two-sample mendelian randomization study. PLoS ONE.

[21] Brion MJ, Shakhbazov K, Visscher PM (2013). Calculating statistical power in mendelian randomization studies. Int J Epidemiol.

[22] Hemani G, Zheng J, Elsworth B, Wade KH, Haberland V, Baird D et al. The MR-Base platform supports systematic causal inference across the human phenome. eLife. 2018;7. 10.7554/eLife.34408 PMC5976434 29846171

[23] Bowden J, Davey Smith G, Haycock PC, Burgess S (2016). Consistent estimation in mendelian randomization with some Invalid Instruments using a weighted median estimator. Genet Epidemiol.

[24] Verbanck M, Chen CY, Neale B, Do R (2018). Detection of widespread horizontal pleiotropy in causal relationships inferred from mendelian randomization between complex traits and diseases. Nat Genet.

[25] Feng T, Malmo V, Laugsand LE, Strand LB, Gustad LT, Ellekjaer H (2020). Symptoms of anxiety and depression and risk of atrial fibrillation-the HUNT study. Int J Cardiol.

[26] Fenger-Gron M, Vestergaard M, Pedersen HS, Frost L, Parner ET, Ribe AR (2019). Depression, antidepressants, and the risk of non-valvular atrial fibrillation: a nationwide danish matched cohort study. Eur J Prev Cardiol.

[27] Fu Y, Feng S, Xu Y, Yang Y, Chen H, He W et al. Association of Depression, Antidepressants with Atrial Fibrillation risk: a systemic review and Meta-analysis. Front Cardiovasc Med. 2022;9. 10.3389/fcvm.2022.897622 PMC9130653 35647056

[28] Gisi B, Althouse AD, Mathier AS, Pusateri A, Rollman BL, LaRosa A (2020). The unmeasured burden: contribution of depression and psychological stress to patient-reported outcomes in atrial fibrillation. Int J Cardiol.

[29] Lu Y, Wang Z, Georgakis MK, Lin H, Zheng L (2021). Genetic liability to Depression and Risk of Coronary Artery Disease, myocardial infarction, and other Cardiovascular Outcomes. J Am Heart Assoc.

[30] Dowlati Y, Herrmann N, Swardfager W, Liu H, Sham L, Reim EK (2010). A meta-analysis of cytokines in major depression. Biol Psychiatry.

[31] Han QQ, Yu J (2014). Inflammation: a mechanism of depression?. Neurosci Bull.

[32] Dantzer R, O’Connor JC, Freund GG, Johnson RW, Kelley KW (2008). From inflammation to sickness and depression: when the immune system subjugates the brain. Nat Rev Neurosci.

[33] Engelmann MD, Svendsen JH (2005). Inflammation in the genesis and perpetuation of atrial fibrillation. Eur Heart J.

[34] Aviles RJ, Martin DO, Apperson-Hansen C, Houghtaling PL, Rautaharju P, Kronmal RA (2003). Inflammation as a risk factor for atrial fibrillation. Circulation.

[35] Palta P, Samuel LJ, Miller ER, Szanton SL (2014). Depression and oxidative stress: results from a meta-analysis of observational studies. Psychosom Med.

[36] Vavakova M, Durackova Z, Trebaticka J (2015). Markers of oxidative stress and neuroprogression in Depression Disorder. Oxid Med Cell Longev.

[37] Barton DA, Dawood T, Lambert EA, Esler MD, Haikerwal D, Hotchkin E (2007). Sympathetic activity in major depressive disorder: identifying those at increased cardiac risk?. J Hypertens.

[38] Sgoifo A, Carnevali L, Alfonso Mde L, Amore M (2015). Autonomic dysfunction and heart rate variability in depression. Stress.

[39] Lu Z, Scherlag BJ, Lin J, Yu L, Guo JH, Niu G (2009). Autonomic mechanism for initiation of rapid firing from atria and pulmonary veins: evidence by ablation of ganglionated plexi. Cardiovasc Res.

[40] Sheng X, Scherlag BJ, Yu L, Li S, Ali R, Zhang Y (2011). Prevention and reversal of atrial fibrillation inducibility and autonomic remodeling by low-level vagosympathetic nerve stimulation. J Am Coll Cardiol.

