# Supplementary material for: Major depression disorder may causally associate with the increased atrial fibrillation risk: evidence from two-sample mendelian randomization analyses
Source: BMC Med Genomics. 2023 Jun 23;16:144. doi: 10.1186/s12920-023-01565-0 (PMC10288724; doi:10.1186/s12920-023-01565-0)
Supplement: Supplementary file 1 — Supplementary Material 1 [file 12920_2023_1565_MOESM1_ESM.doc]

Supplemental Material

| SNP | Effect_allele | Other_allele | Chr | Position | EAF | beta | se |
| --- | --- | --- | --- | --- | --- | --- | --- |
| rs7551758 | G | T | 1 | 52274078 | 0.5329 | 0.0283 | 0.0043 |
| rs2568958 | A | G | 1 | 72765116 | 0.6042 | 0.0382 | 0.0044 |
| rs10913112 | T | C | 1 | 175913828 | 0.378 | -0.0262 | 0.0045 |
| rs17641524 | T | C | 1 | 197704717 | 0.2101 | -0.03 | 0.0053 |
| rs354155 | C | G | 1 | 49675276 | 0.0923 | -0.0449 | 0.0075 |
| rs7538938 | C | T | 1 | 67132262 | 0.5599 | 0.0251 | 0.0043 |
| rs4141983 | C | T | 1 | 18122009 | 0.326 | -0.0264 | 0.0046 |
| rs2111592 | A | G | 2 | 208049581 | 0.3141 | 0.0263 | 0.0046 |
| rs72948506 | A | G | 2 | 212618440 | 0.2975 | 0.0265 | 0.0047 |
| rs35469634 | G | A | 3 | 158171455 | 0.5774 | -0.0241 | 0.0044 |
| rs843812 | A | G | 3 | 61255413 | 0.4117 | 0.0248 | 0.0044 |
| rs9831648 | T | G | 3 | 49214303 | 0.7739 | -0.0292 | 0.0052 |
| rs66511648 | C | T | 3 | 117515519 | 0.284 | 0.0297 | 0.0048 |
| rs76954012 | A | T | 3 | 115977242 | 0.0931 | 0.0412 | 0.0074 |
| rs30266 | A | G | 5 | 103972357 | 0.3271 | 0.0366 | 0.0046 |
| rs247910 | G | A | 5 | 87630769 | 0.457 | 0.0237 | 0.0043 |
| rs7725715 | A | G | 5 | 164487555 | 0.5343 | 0.029 | 0.0043 |
| rs150186873 | C | A | 6 | 27182377 | 0.0327 | 0.0704 | 0.012 |
| rs2232423 | G | A | 6 | 28366151 | 0.1056 | -0.062 | 0.007 |
| rs9364755 | G | A | 6 | 165117329 | 0.2262 | 0.0283 | 0.0051 |
| rs2214123 | G | A | 6 | 67000001 | 0.6466 | -0.0261 | 0.0045 |
| rs2876520 | G | C | 6 | 142996618 | 0.4688 | 0.026 | 0.0043 |
| rs2522831 | C | T | 7 | 82448100 | 0.4739 | 0.024 | 0.0043 |
| rs4730387 | A | T | 7 | 109100414 | 0.4659 | 0.0238 | 0.0043 |
| rs150346963 | T | C | 7 | 117625599 | 0.4118 | 0.0283 | 0.0044 |
| rs3807865 | A | G | 7 | 12250402 | 0.4105 | 0.031 | 0.0044 |
| rs10235664 | C | T | 7 | 2086814 | 0.2529 | -0.027 | 0.0049 |
| rs59082935 | T | C | 7 | 38724868 | 0.1342 | 0.0363 | 0.0066 |
| rs62535714 | A | G | 9 | 37182655 | 0.1639 | 0.0339 | 0.0058 |
| rs1931388 | G | A | 9 | 11203149 | 0.4042 | -0.0295 | 0.0044 |
| rs59283172 | A | G | 9 | 25232978 | 0.1081 | -0.039 | 0.007 |
| rs2418449 | C | T | 9 | 119731359 | 0.281 | -0.0281 | 0.0048 |
| rs1021363 | G | A | 10 | 106610839 | 0.6434 | -0.03 | 0.0045 |
| rs198457 | T | C | 11 | 61471678 | 0.1886 | -0.0315 | 0.0056 |
| rs4497414 | C | T | 11 | 88756779 | 0.44 | 0.0291 | 0.0044 |
| rs4936276 | C | G | 11 | 113365141 | 0.622 | 0.0278 | 0.0044 |
| rs61914045 | A | G | 12 | 52352301 | 0.2034 | 0.0309 | 0.0054 |
| rs9529218 | T | C | 13 | 31790053 | 0.2031 | -0.034 | 0.0054 |
| rs9536381 | T | C | 13 | 53860655 | 0.3259 | 0.0255 | 0.0046 |
| rs508502 | T | C | 13 | 80921519 | 0.2992 | -0.0264 | 0.0048 |
| rs1950829 | G | A | 14 | 42097937 | 0.5173 | -0.0297 | 0.0043 |
| rs754287 | A | T | 14 | 103997525 | 0.3664 | -0.0289 | 0.0045 |
| rs7152906 | C | T | 14 | 75125540 | 0.5196 | 0.0258 | 0.0043 |
| rs28541419 | G | C | 15 | 88945878 | 0.2308 | -0.0292 | 0.0052 |
| rs12919291 | C | G | 16 | 13800430 | 0.1884 | 0.0327 | 0.0055 |
| rs4799949 | T | C | 18 | 35155910 | 0.6684 | -0.0292 | 0.0046 |
| rs12967143 | C | G | 18 | 53099012 | 0.7012 | -0.0345 | 0.0047 |
| rs7241572 | A | G | 18 | 77580712 | 0.2047 | 0.0323 | 0.0054 |
| rs1367635 | C | T | 18 | 50861409 | 0.5148 | 0.0253 | 0.0043 |
| rs13037326 | T | C | 20 | 44692598 | 0.2597 | 0.031 | 0.0049 |

Table S1. The information on the 50 selected SNPs with major depressive disorder.

Abbreviations: SNP single nucleotide polymorphism, Chr chromosome, EAF effect allele frequency, se standard error.
